# Editorial: Coupling and Uncoupling: Dynamic Control of Membrane Contacts

**DOI:** 10.3389/fcell.2021.721546

**Published:** 2021-08-24

**Authors:** Dan Zhang, Yasunori Saheki, Junjie Hu, Benoît Kornmann

**Affiliations:** ^1^Temasek Life Sciences Laboratory, National University of Singapore, Singapore, Singapore; ^2^Department of Biological Sciences, National University of Singapore, Singapore, Singapore; ^3^Lee Kong Chian School of Medicine, Nanyang Technological University, Singapore, Singapore; ^4^Institute of Resource Development and Analysis, Kumamoto University, Kumamoto, Japan; ^5^National Laboratory of Macromolecules, Institute of Biophysics, Chinese Academy of Sciences, Beijing, China; ^6^College of Life Sciences, University of Chinese Academy of Sciences, Beijing, China; ^7^Department of Biochemistry, University of Oxford, Oxford, United Kingdom

**Keywords:** membrane contact sites, organelle dynamics, membrane-cytoskeleton interactions, membrane contact remodeling, non-vesicular lipid transport

All living cells are dynamic assemblies in nature that continuously self-organize numerous molecules into various separate-yet-connected functional and homeostatic units. Many such membrane-bound units in eukaryotic cells, namely membranous organelles with characteristic morphologies, dynamics, and functions, together with the plasma membrane (PM) are physically interconnected at membrane contact sites (MCSs) without membrane fusion. MCSs are commonly regarded as interorganellar, though they can also be intraorganellar. To date, all membrane-bound organelles have been observed to make contacts with at least one other membrane type. The distribution and abundance of these interorganellar MCSs vary among cell types. Structurally, some MCSs could appear stable and constitutive [e.g., the endoplasmic reticulum (ER)-PM contacts in muscle cells and yeasts, etc]. However, membrane tethering as well as molecular assemblies at MCSs are often highly dynamic. For example, transient contacts between the ER and mitochondria (Mito) or endosomes (Endo) form during their respective fission. In mammalian cells, machineries for store-operated Ca^2+^ entry are rapidly assembled at ER-PM contacts upon reduction of Ca^2+^ levels in the ER lumen.

Close membrane apposition can be established in both an inter- and intraorganellar fashion through a variety of tethering machineries. Interorganellar tethering complexes facilitate direct transport of small molecules, such as lipids and ions at MCSs, which helps to maintain cellular homeostasis and guide associated signaling pathways and/or cellular events. Intraorganellar tethers on the other hand can support the architecture of organelles and hence their functions: They act either at the cytoplasmic interface to build stacked membranes [e.g., stacked ER cisternae albeit more prominently seen with the overexpression of ER resident proteins (Snapp et al., 2003) and Golgi stacks (Lee et al., 2014) etc.], or as intraluminal spacers to modulate lumen diameter [e.g., ER luminal spacer Climp63 (Zhao and Hu)], or in a complex manner to structure the interior of double-membrane-bound organelles, such as mitochondria (van der Laan et al., 2012). In all cases, spatial confinement at these membrane junctures could locally alter molecular dynamics and potentially involve microdomain formation for specialized activities.

With the discovery of major tethering complexes for various MCSs, one of the next major goals is to understand how cells spatiotemporally control assembly and disassembly of MCSs and coordinate the functions of various MCSs during different states of the cells. Many tethers have been pinpointed to couple membranes through protein-protein or protein-lipid interaction, however little is known quantitatively about the threshold of such interactions that suffices bona fide contact site formation. This is an important issue, as cross-membrane interactions essentially determine the membrane tethering strength and therefore could impact the abundance and dynamics of MCSs. Conceivably, modulation of such interactions at MCSs is likely the common mechanism for dynamic control of MCS formation in the cell. Thus, one should be cautious in interpreting findings that involve tethering proteins expressed at non-physiological levels for functional indication, as higher levels of tethers often induce the formation of ectopic and/or more stable MCSs, and vice versa, which may alter their functions. Intrinsic membrane remodeling capacity and cytoskeleton dynamics (Koppers et al.) could also affect the strength of membrane tethering at MCSs, possibly through imposing shear forces or cross-linking effects. Besides, organelle morphology, particularly the ER reticulation, appears crucial for MCS formation: For instance, membrane contacts are more frequently engaged with the tubular ER. Alteration in membrane curvature or geometry during organellar remodeling may locally promote the coupling or uncoupling between membranes.

Limited spacing at MCSs could obstruct space-demanding processes that involve large-sized protein assemblies (Zhang, 2020) or vesicles (Ng et al., 2018). It is conceivable that such physical roles would depend on the amount and plasticity of MCSs, and hence the disassembly and removal of MCS become an important step to finetune this barrier effect. While as for biochemical activities at MCSs, it is becoming clear that tethering of opposing membranes stimulates non-vesicular lipid transport mediated by lipid-transfer proteins (LTPs) (Ercan et al., 2021). How the assembly and disassembly of MCSs affect the global state of lipid transport, however, remains elusive. In fact, many LTPs have affinities for both membranes at MCSs through the binding of lipids or resident proteins, so they are often considered as part of the tethering machinery. However, most LTPs seem to have minor roles in sustaining MCSs under normal physiological conditions. One can envisage that non-vesicular lipid transfer could also occur via simple diffusion of LTPs following the lipid gradient between membranes. Indeed, some forms of non-vesicular lipid transport likely do not rely on MCSs (Dittman and Menon, 2017; Quon et al., 2018). Nevertheless, commonality of microniches created by juxtaposed membranes in lipid-related functions, including lipid transport, synthesis, and metabolism (discussed by Hewlett et al.; Nakatsu and Kawasaki; Xu and Huang), infers that MCSs should possess conserved advantages for these roles.

In this collection of articles, we see the latest summaries and perspectives on MCSs of various kinds covering their formation, dynamics, and functions, including ER-PM (Hewlett et al.; Li et al.), ER-Mito (Aoyama-Ishiwatari and Hirabayashi), ER-Endo (Shirane), ER-Golgi (Feng et al.), Mito-lipid droplet (LD) (Cui and Liu), and secretory vesicle-PM contacts (Ruan et al.), etc. In addition, Tashiro et al. developed an improved version of the split-GFP system in yeast and mammalian cells (which relies on lower expression of the split-GFP components), enabling quantitative analysis of various MCSs at near-native states in living cells. Cui and Liu further discuss potential molecular distinctions between dynamic and stable Mito-LD contacts from a structural point of view. Moreover, two articles particularly highlight anomalies of MCSs in various diseases, e.g., altered abundance of ER-Mito contacts being seen in different cell lineages associated with cardiovascular diseases (Liu et al.) and impaired endosomal homeostasis resulting from ER-Endo contact dysfunction perhaps underlying some forms of neurodegenerative disorders (Shirane). Finally, Wong et al. draw an interesting connection between host cell organelles and viral RNA-containing double-membrane vesicles (DMVs) for positive strand RNA viral replication, using severe acute respiratory syndrome coronavirus 2 as a major example. They review how these viruses hijack host lipid metabolic machineries and redirect lipids via assorted MCSs to generate DMVs for proliferation.

## Unfathomed Questions and Challenges

Membrane-contact-related research has gained tremendous interest in recent years. While exciting advances have been made, many fundamental questions remain elusive. These include:

- How is the rate of non-vesicular lipid transport regulated in living cells? So far, lipid transfer has been primarily studied using reconstitution assays, but the rate of lipid transport observed *in vitro* is often slow. It is also intriguing to see how much of non-vesicular lipid transport is mediated at MCSs vs. by simple diffusion of LTPs *in vivo*. Further attempts will have to be made to establish methods for detecting and quantifying such lipid transfer in living cells.
- How does redundancy of multiple protein tethers contribute to their functional crosstalk at the same MCSs? The abundance and structural arrangement of individual tethers likely matter, especially when they differ in distances that they can stretch at MCSs. This question is pertinent to a common issue that many studies use depletion or over-expression of tethers to argue their functions. With potential interference among tethers (i.e., depletion or overexpression of tether A affecting tether B functions), we may need to re-evaluate some of the existing results.
- How does molecular interaction physically determine the extent of membrane tethering at MCSs? Addressing this question will provide mechanistic insights into the dynamic control of MCS remodeling.
- A general one–how do cells balance different MCSs in their quantity, distribution, dynamics, and functions, in harmony with their cellular states?

## Author Contributions

All authors listed have made a substantial, direct and intellectual contribution to the work, and approved it for publication.

## Conflict of Interest

The authors declare that the research was conducted in the absence of any commercial or financial relationships that could be construed as a potential conflict of interest.

## Publisher's Note

All claims expressed in this article are solely those of the authors and do not necessarily represent those of their affiliated organizations, or those of the publisher, the editors and the reviewers. Any product that may be evaluated in this article, or claim that may be made by its manufacturer, is not guaranteed or endorsed by the publisher.
